# Microbial Community Composition Associated with Potato Plants Displaying Early Dying Syndrome

**DOI:** 10.3390/microorganisms13071482

**Published:** 2025-06-26

**Authors:** Tudor Borza, Rhea Amor Lumactud, So Yeon Shim, Khalil Al-Mughrabi, Balakrishnan Prithiviraj

**Affiliations:** 1 Department of Plant, Food and Environmental Sciences, Faculty of Agriculture, Dalhousie University, 50 Pictou Road, Cox Institute, Truro, NS B2N 5E3, Canada; rheaamor@dal.ca; 2 School of Climate Change and Adaptation, University of Prince Edward Island, 550 University Avenue, Charlottetown, PE C1A 4P3, Canada; sshim@upei.ca; 3 Department of Agriculture, Aquaculture and Fisheries, 39 Barker Lane, Wicklow, NB E7L 3S4, Canada; khalil.al-mughrabi@gnb.ca

**Keywords:** potato early dying, endosymbionts, *Verticillium dahliae*, amplicon-targeted next-generation sequencing

## Abstract

Potato early dying disease complex (PED) leads to premature senescence and rapid decline in potato plants. Unlike potato wilt caused solely by *Verticillium* species, PED symptoms are more severe due to the synergistic effects of multiple pathogens, including root-lesion nematodes, fungi such as *Colletotrichum* and *Fusarium*, and soft-rot bacteria. To investigate the microbiome responsible for PED, soil and stem samples from healthy-looking and symptomatic plants were analyzed using amplicon-targeted next-generation sequencing (Illumina MiSeq and PacBio technologies). Samples were collected from four locations in New Brunswick, Canada from fields previously rotated with barley or oat. Comparative analysis of the bacterial, fungal, and eukaryotic diversity in soil samples showed minimal differences, with only bacterial alpha diversity influenced by the plant health status. *Verticillium dahliae* was abundant in all soil samples, and its abundance was significantly higher in the stems of diseased plants. Additional fungal species implicated in PED, including *Plectosphaerella cucumerina*, *Colletotrichum coccodes*, *Botrytis* sp., and *Alternaria alternata*, were also identified in the stems. This study highlights the complex, plant-associated microbial interactions underlying PED and provides a foundation for microbiome-informed disease management strategies.

## 1. Introduction

Potato early dying disease complex (PED) is a significant potato disease affecting major production areas worldwide. It causes yield reduction, having a substantial economic impact. Potato plants with PED undergo premature senescence, followed by rapid demise [1,2]. The primary pathogens responsible for PED belong to the *Verticillium* genus, particularly species causing wilt in potatoes. These include *Verticillium dahliae* Kleb, *V*. *nonalfalfae* Inderb et al., and *V*. *albo-atrum* Reinke & Berthold. [3]. While *Verticillium* species generally cause less severe symptoms than PED, the cumulative effects of other pathogens, such as root-lesion nematodes (genus *Pratylenchus*), fungi (*Colletotrichum* and *Fusarium*), soft-rot bacteria, and abiotic stresses (e.g., nutrient deficiencies), exacerbate the disease [2].

In the last decade, the Maritime region of Canada (New Brunswick, Prince Edward Island, and Nova Scotia) has seen an increase in PED symptoms and a sharp decline in marketable tuber yields. The presence and severity of *Verticillium* in potato fields in this region have been assessed thoroughly in the last decade [4,5,6], indicating that *V. dahliae* is prevalent in potato fields in these provinces. Additionally, a recent survey reported on root-lesion nematode populations in New Brunswick and Prince Edward Island [6].

Soil microbiomes have complex interactions, and PED results from such interactions. Characterizing the composition of these soils and estimating the cumulative effect of these complex interactions among microbes is crucial to understand the occurrence of complex diseases. These approaches also allow understanding if and how soil promotes disease suppression or fosters disease development. Interactions among different microbial species can enhance or inhibit the overall disease-suppressive potential of the soil [7,8]. Assessing microbiome diversity across fields used in potato cropping systems allows characterization of pathogen diversity, abundance, and a better understanding of the variation in pathogen suppressive populations [9]. Next-generation sequencing of potato soil microbiomes (bacterial and eukaryotic communities) in nine U.S. fields showed a consistent phylum-level composition across locations, with regional differences among genera and amplicon sequence variants [10]. This study also identified a complex core community that included common soil genera like *Bacillus* and *Mortierella*.

Assessing soil microbiome diversity is important, but explaining the effects on disease development can be difficult, considering the diversity of the microbiome and the complex interactions that take place at this level. Characterizing communities that are associated directly with plants is equally important. Pathogen–plant interaction as well aspathogen colonization and development in plants can be promoted or inhibited by endophytic microbiota. Several studies have explored the antagonistic effects of various endophytes on potato pathogens. Reiter et al. [11] found that potatoes infected with *Pectobacterium atrosepticum* (formerly *Erwinia carotovora* subsp. *atroseptica*) increased the endophyte (bacterial) diversity in infected plants; a few of these endophyte isolates were antagonistic against blackleg disease. The antagonistic effect of several bacterial endophytes on *P. atrosepticum* was also reported by Pavlo et al. [12], who showed that endophytes have the potential to activate plant defense systems. Bahmani et al. [13] found that several potato endophytic bacteria have antagonistic effects against *Ralstonia solanacearum*, which causes potato wilt. Analysis of the composition and relative abundance of endophytic fungi in the roots of potatoes by Götz et al. [14] revealed the presence of many species, with the most frequently isolated species being *V. dahliae*, *Cylindrocarpon destructans*, *Colletotrichum coccodes*, and *Plectosporium tabacinum* (the anamorph of *Pectospherella cucumerina*). The antagonistic effects of some potato endophytes are not limited to bacteria and fungi, as Sorokan et al. [15] found that the endophytic strain *Bacillus subtilis* 26D promotes the mortality of the potato beetle *Leptinotarsa decemlineata.*

To contribute to the elucidation of the complexities related to PED, it is essential to characterize both soil microbiota and endophyte communities. Therefore, with this aim, the composition of soil microbiota in the proximity of diseased- and healthy-looking plants, as well as the composition of endophytes colonizing the stems of these plants, from four fields cultivated with potatoes in the province of New Brunswick, Canada, was assessed through amplicon-targeted next-generation sequencing.

## 2. Materials and Methods

### 2.1. Sample Collection and Sampling Location

Sample collection took place in early September at four potato-cultivated fields: two located in the Bloomfield area and two in the Knoxford area of New Brunswick, Canada. In the previous year, the rotation crops in the Bloomfield fields were oats, while in the Knoxford fields, they were barley. From each field, 10 stem samples from healthy-looking plants and from plants showing signs of PED (i.e., diseased-looking plants) were randomly collected (Figure S1). The stems, which had a maximum diameter of 2 cm and were 4–6 cm long, were cut from the lower parts of the plants roughly 10 cm from the ground and placed in a Ziploc^®^ plastic bag. Soil samples from the proximity of these plants were collected using a Dutch auger and mixed, and subsets were placed in 50 mL vials. Meanwhile in the field, samples were placed in a cooler filled with ice packs and then stored frozen (−20 °C) until processing.

### 2.2. Soil Type

The soil in the area from where the samples were collected is silty clay loam to silt loam with >20% clay. The cation exchange capacity (CEC) in the region is 13–19, while the pH is maintained close to 6. The organic matter percentage ranges from 3.5 to 6%.

### 2.3. Sample Processing and DNA Extraction

After sampling, the plastic bags containing the stems were maintained at −20 °C. In the laboratory, the frozen stems were briefly washed with distilled water and then with a solution of 0.1% bleach. A layer of approximately 1 mm, comprising the epidermis and the superficial part of the cortex, was removed. Slices of 100–200 mg from each stem were then cut into small pieces, mixed, and placed in individual 1.5 mL tubes that were maintained at −20 °C until used for DNA isolation. Stem DNA isolation was performed using 100 mg of plant tissue and a GenJET™ Plant Genomic DNA purification kit (Fisher Scientific, Toronto, ON, Canada) as recommended by the manufacturer’s protocol. DNA was eluted in 100 µL of buffer, and the DNA concentration was estimated using a nanodrop (NanoDrop 200, Thermo Fisher Scientific, Waltham, MA, USA). DNA extraction from soil samples was performed using 250 mg of soil and a PowerSoil^®^ DNA Isolation Kit (MoBio Laboratories, Inc., Carlsbad, CA, USA). Samples were homogenized using a Mini BeadBeater^TM^ cell disruptor (BioSpec Products, Inc., Bartlesville, OK, USA). DNA was eluted in 100 µL of buffer, and the DNA concentration was estimated as described above.

### 2.4. Quantitative Real-Time Polymerase Chain Reaction

Identification and quantification of *Verticillium* DNA from plant and soil samples were performed by qPCR using a StepOnePlus Real-Time PCR system (Applied Biosystems, ThermoFisher Scientific, Burlington, ON, Canada). qPCR was performed in a 20 µL reaction volume using 2 µL of DNA (2–5 ng DNA), 1 µL 4 μM each of the forward and reverse primers [4,16], and 10 µL 2X iTaq™ Universal SYBR^®^ Green Supermix (BioRad, Mississauga, ON, Canada). The cycling parameters consisted of one denaturing cycle of 95 °C for 30 s, followed by 40 cycles of 95 °C for 10 s and 60 °C for 30 s. The absolute quantification (standard curve) method was used to determine the amount of *V. dahliae* in the plant and soil samples. DNA from *V. dahliae* isolate Vs04-41 [17,18] was used as a standard. Quantification was performed by comparing the data with triplicate DNA standards (five 1:5 serial dilutions covering the range from 0.2 ng to 0.32 pg) as described by Borza et al. [4]. The amount of DNA in the target samples and the amplification efficiency were determined automatically using StepOnePlus^TM^ software, v2.3. Calculations of the amount of DNA corresponding to one *V. dahliae* cell were performed while considering 36.5 fg/genome as described elsewhere [4,19], based on a *V. dahliae* genome size of 33.8 Mb [20].

### 2.5. Stem and Soil Sample Amplicon-Targeted Next-Generation Sequencing

Samples have been sequenced using two different amplicon-targeted next-generation sequencing methods, i.e., Illumina MiSeq technology (NGSI) and PacBio technology (NGSPB). Sequencing and initial data analysis were performed using the Integrated Microbiome Resource (IMR) platform at Dalhousie University, NS, Canada.

All samples were sequenced using NGSI, and the targets were 16S, 18S, and ITS2 (Table S1). The amplicon size was 380 nucleotides (nt) for 16S, 360 nt for 18S, and 260 nt for ITS. NGSI generates rather short amplicons; therefore, it was used to better understand the dynamics of the assemblage of microbiota, including other potential soil-borne pathogens responsible for PED, in addition to screening for the pathogenic *Verticillium* species and nematodes.

NGSPB generates longer amplicons, which allow a more precise taxa identification. To improve identification at the genus and, if possible, species levels of the taxa that were likely to be responsible for PED, half of the samples sequenced using the NGSI were re-sequenced using the NGSPB. The targets were 16S and ITS2 (Table S2), and the amplicon size was >1000 nt and >500 nt, respectively. Amplicon-targeted next-generation sequencing data were submitted to the Sequence Read Archive (SRA) (project ID: PRJNA1263883; SRA study SRP585969; BioSamples accessions SAMN48542972–SAMN48543131).

### 2.6. Microbiome Workflow and Data Analysis

Raw sequencing reads were processed using a QIIME 2-based [21], IMR-developed pipeline [22]. Sequence quality control, denoising, and chimera removal were performed using Deblur [23]. We applied filtering criteria to remove low-abundance taxa. Specifically, taxa were retained in the dataset with a minimum read count of 4 in at least 5% of the samples. Soil bacterial samples with library sizes < 2000 were not included in downstream analyses. The library sizes that were included in downstream analyses ranged from 2104 to 18,361 reads, with an average of 9240 reads. For the stem bacterial samples, library sizes ranged from 122 to 1279 reads with an average of 736 reads. For the soil bacteria, 3691 amplicon sequence variants (ASVs) were generated from 76 samples, while 175 ASVs were generated for stems from 8 samples. For the soil fungi, library sizes ranged from 6902 to 130,353 reads, with an average size of 35,070 reads, generating 844 ASVs from 75 samples. For the stem fungi, library sizes < 1000 reads were filtered out, resulting in library sizes ranging from 1023 to 59,749 reads, with an average size of 9510 reads, generating 41 ASVs across 46 samples. Features and representative sequences were then constructed using the SILVA (for 16S and 18S data) and UNITE (for ITS2 data) databases.

The alpha and beta diversity metrics were analyzed using QIIME 2. Alpha diversity was calculated using the Shannon index, Chao1, and observed features and ASVs. Beta diversity was calculated using the Bray–Curtis dissimilarity and weighted and unweighted UniFrac distances and visualized through principal coordinate analysis (PCoA). Differential abundance analysis was conducted using EdgeR [24,25]. ASV count data were normalized using the trimmed mean of M values (TMM) method. Differentially abundant taxa between healthy and diseased samples were identified using a negative binomial generalized log-linear model, with significance determined at an FDR-adjusted *p* value < 0.05. Heatmaps were generated using the ComplexHeatmap (v2.20.0) package in R. The log2-transformed abundance data were scaled, and hierarchical clustering was applied. All R-based analyses were performed in R Studio (v2024.09.0+375) running R (v4.4.0).

The NGSI dataset was further used to analyze microbiome network co-occurrence and differential structures related to plant health status. Fungal interaction networks at the genus level were constructed for stem samples using the SPIEC-EASI R package v1.1.3 (spiec.easi function) with the Meinshausen–Buhlmann neighborhood selection method [26]. The resulting adjacency matrix of conditional dependencies was converted into a graph object using igraph::adj2igraph() [27]. Node sizes corresponded to the mean relative abundances, while genera were differentiated using pastel colors via RColorBrewer. Edge colors indicated positive (red) and negative (blue) associations based on partial correlations. To characterize and compare the topology of healthy and diseased fungal networks, several structural metrics were computed using the igraph package. The Jaccard index quantified the overlap between healthy and diseased fungal communities. Hub genera were identified based on degree centrality.

Differential abundance analysis used linear discriminant analysis effect size (LEfSe) [28] via the microeco package [29]. The phyloseq2meco() function from microeco was used to convert the phyloseq object into a format compatible with LEfSe analysis. The trans_diff$new() function was then applied to perform the LEfSe test, using the health status of the plant (healthy vs. diseased) as the grouping variable. Significantly different genera were identified with an LDA score > 2.0 (*p* = 0.05). Results were visualized as cladograms depicting taxonomic relationships from phylum to genus, highlighting significant biomarkers by health status.

## 3. Results

### 3.1. Soil Microbiota Around Healthy- and Diseased-Looking Potato Plants

#### 3.1.1. *V. dahliae* Incidence and Abundance in Soil Collected from the Proximity of Healthy- and Diseased-Looking Potato Plants

The qPCR analyses of *V. dahliae* incidence and abundance in the soil samples showed no significant differences (*p* > 0.05; two-sample *t*-test) between samples collected from the proximity of healthy- and diseased-looking plants in any of the fields sampled (Table 1). *V. dahliae* was found in all soil samples sequenced using NGSI, while NGSPB sequencing detected *V. dahliae* in 82% (32 out of 39 successful sequencing reactions) of the soil samples collected around either healthy- or diseased-looking plants.

#### 3.1.2. Bacterial Diversity and Abundance in Soil Samples Collected from the Proximity of Healthy- and Diseased-Looking Potato Plants

**Figure 1:**
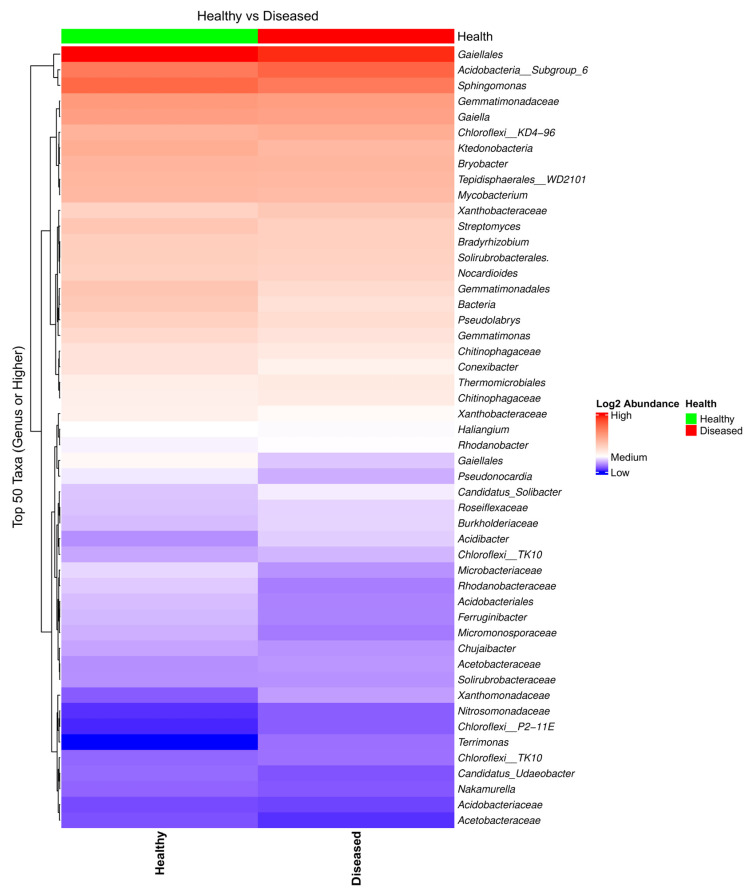
Heatmap of the bacterial taxa composition in soil samples collected from the proximity of diseased- and healthy-looking potato plants. NGSI data.

Analysis of the heatmap and the bar plot showing the bacterial taxa composition in the soil samples collected from the proximity of diseased- and healthy-looking plants revealed a high abundance of taxa, with a limited number of differences between the two conditions (Figure 1 and Figure S2).

Shannon’s alpha diversity of bacterial biota from soil samples collected from the proximity of healthy- and diseased-looking plants, analyzed using a Kruskal–Wallis pairwise comparison test, revealed differences that were statistically significant (*p* = 0.00598). Beta diversity analyses, on the other hand, revealed differences related to the health status of the plant that were less obvious (Figure S3).

#### 3.1.3. Fungal Diversity and Abundance in Soil Samples Collected from the Proximity of Healthy and Diseased-Looking Potato Plants

**Figure 2:**
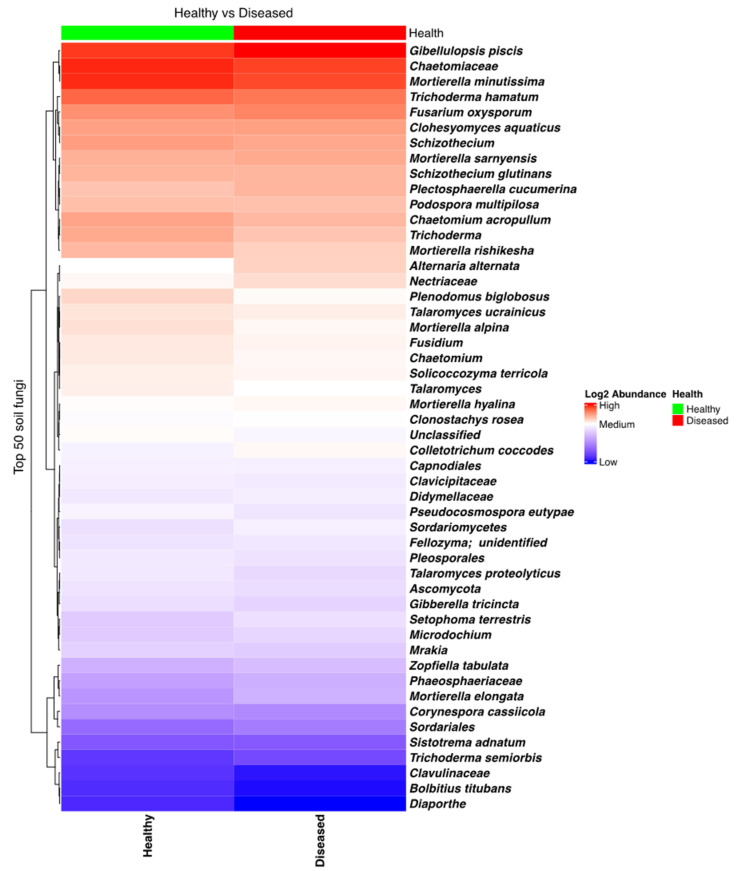
Heatmap of the fungal taxa composition in soil samples collected from the proximity of diseased- and healthy-looking potato plants. NGSI data.

The fungal community in soil found in the proximity of both diseased and healthy plants was rich and diverse, with many taxa present. In particular, taxa such as *Trichoderma*, *Mortierella*, and *Gibellulopsis piscis* (=*Gibellulopsis nigrescens*, former *Verticillium nigrescens*) [30,31] were most frequent (Figure S4). Several other genera, comprising species that influence potato health, were also found to have high relative frequencies in several samples, such as *Verticillium*, *Fusarium*, *Colletotrichum*, *Alternaria*, and *Gibellulopsis*. A heatmap analysis showed that *Fusarium oxysporum*, which can cause wilting and dry rot in potatoes, was quite abundant in all soil samples, while *Alternaria alternata*, which causes brown spots on potatoes, was found to be more prevalent in the soil samples collected close to the diseased plants. An opposite trend, being more abundant in soil close to healthy plants, was observed in the case of another potato pathogen, namely *C. coccodes*, which is responsible for black dot disease (Figure 2). However, none of these differences were found to be statistically significant.

No significant differences were found when analyzing Shannon’s alpha diversity indices of the soil samples from the proximity of healthy- and diseased-looking plants (*p* = 0.61818). Similarly, the beta diversity plot, generated using the weighted UniFrac distance metric, showed no substantial differences in relation to plant health (Figure S5).

As mentioned in Section 3.1.1, NGSPB sequencing revealed that 82% (32 out of 39 successful sequencing reactions) of the soil samples, collected around either healthy- or diseased-looking plants, contained *V. dahliae*. Among common soil saprotrophs, several species of *Mortierella*, *Trichoderma*, and *Penicillium* have been identified as being highly abundant. Among plant fungal pathogens, including potato pathogens, *Botrytis* was found to be particularly abundant, followed by *A. alternata* and *C. coccodes*. Other fungal pathogens which were found in most soil samples and relatively abundant were *F. oxysporum*, *F. graminearum*, *F. solani*, *V. albo-atrum*, and *P. cucumerina*.

Shannon’s alpha diversity indices indicated no significant differences (*p* = 0.500094) when the two different types of soil samples were compared. The beta diversity plot, generated using the weighted UniFrac distance metric, showed no substantial differences in relation to plant health.

#### 3.1.4. Eukaryote Diversity and Abundance in Soil Samples Collected from the Proximity of Healthy- and Diseased-Looking Potato Plants

**Figure 3:**
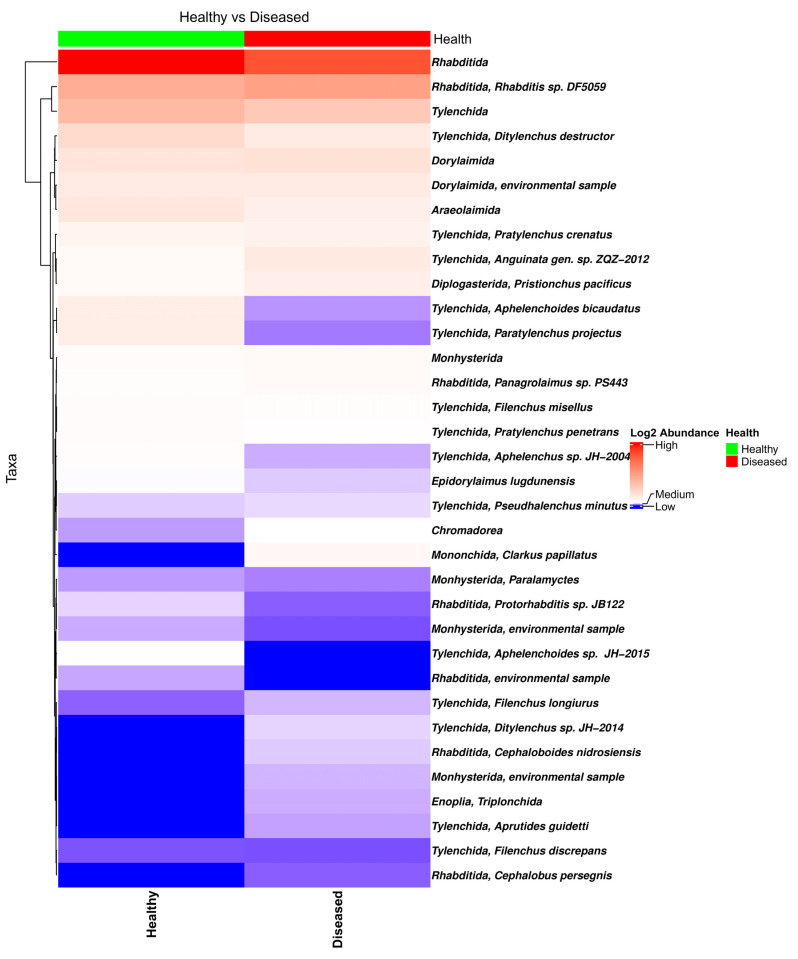
Heatmap of the nematode composition in soil samples collected from the proximity of diseased- and healthy-looking potato plants. NGSI data.

The bar plot of the relative frequency of the eukaryotic taxa in the soil samples collected from the proximity of healthy- and diseased-looking plants showed no major differences (Figure S6). Among the eukaryote taxa identified in the data, of special interest is the large number of nematode taxa from the orders Tylenchida (genera *Pratylenchus*, *Ditylenchus*, *Filenchus*, and *Helicotylenchus*) and Rhabditida (genera *Rhabditis*, *Cephalobus*, and *Distolabrellus*) (Figure 3), fungi (genus *Verticillium*), and Oomycetes (genera *Phytophthora* and *Pythium*).

A Kruskal–Wallis pairwise comparison test on Shannon’s alpha diversity indices of eukaryote biota in the soil samples showed no significant differences between the soil samples collected from the proximity of healthy- and diseased-looking plants (*p* = 0.6738). Similarly, the beta diversity plot revealed limited differences between the two types of samples, suggesting that plant health is not associated with dissimilarities between the soil eukaryote communities found in the proximity of healthy and diseased plants.

### 3.2. Microbiota Associated with the Stems of Healthy- and Diseased-Looking Potato Plants

#### 3.2.1. *V. dahliae* Incidence and Abundance in the Stems of Healthy and Diseased Potato Plants

The incidence of *V. dahliae* in plant samples (stems) collected from healthy and diseased-looking plants was high, but no differences were found between this parameter and the health status of the plant (Table 2). However, the abundance of *V. dahliae* in the stems from diseased plants was significantly higher, in some cases being more than an order of magnitude, and this difference was statistically significant. A similar situation was observed in the NGSI data targeting the fungal region ITS2. Based on NGSI data, the incidence of *V. dahliae* in the stem samples was 100% in all conditions (health status and fields), except for 90% in the healthy potato plant samples collected from fields in which the previous year’s crop was barley. The significance of differences between the healthy- and diseased-looking plants was borderline when using edgeR; that is, *p* = 0.0173, but FDR = 0.0664.

Similar to NGSI, NGSPB sequencing found that all stems, either from healthy- or diseased-looking plants, contained large amounts of *V. dahliae*. In each location, the number of reads/sample (number of DNA molecules sequenced/sample) corresponding to *V. dahliae* were higher (on average >10 times higher) in the stems from diseased-looking plants compared with the healthy-looking plants. edgeR revealed that these differences were statistically significant, considering both parameters (*p* = 0.0002 and FDR = 0.0036).

#### 3.2.2. Bacterial Diversity and Abundance in the Stems of Healthy- and Diseased-Looking Potato Plants

**Figure 4:**
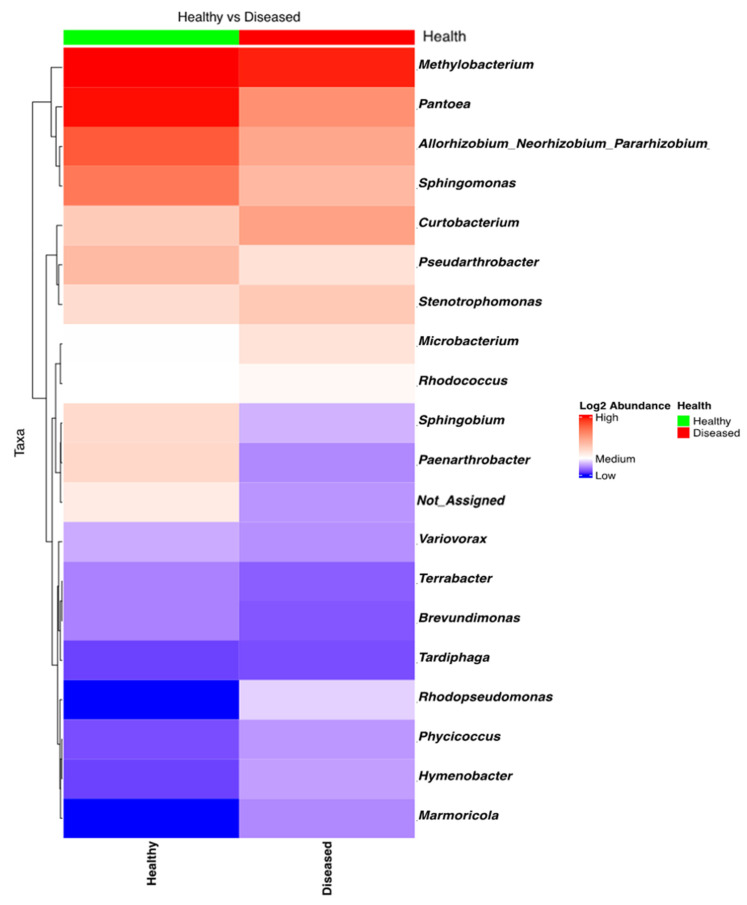
Heatmap of the bacterial taxa found in the potato stems of diseased- and healthy-looking plants. NGSI data.

NGSI data targeting the 16S amplicon was strongly influenced by the low success rate (10%) of sequencing and data retrieval; ASV data could be generated only for 8 out of 80 samples, which gave only 98 ASVs (Figure 4). Shannon’s alpha diversity of the eight bacterial samples (four from diseased-looking plants and four from healthy-looking plants) was analyzed using a Kruskal–Wallis pairwise comparison test, which indicated that there were no significant differences between the two groups (*p* = 0.7728).

#### 3.2.3. Fungal Diversity and Abundance in the Stems of Healthy- and Diseased-Looking Potato Plants

**Figure 5:**
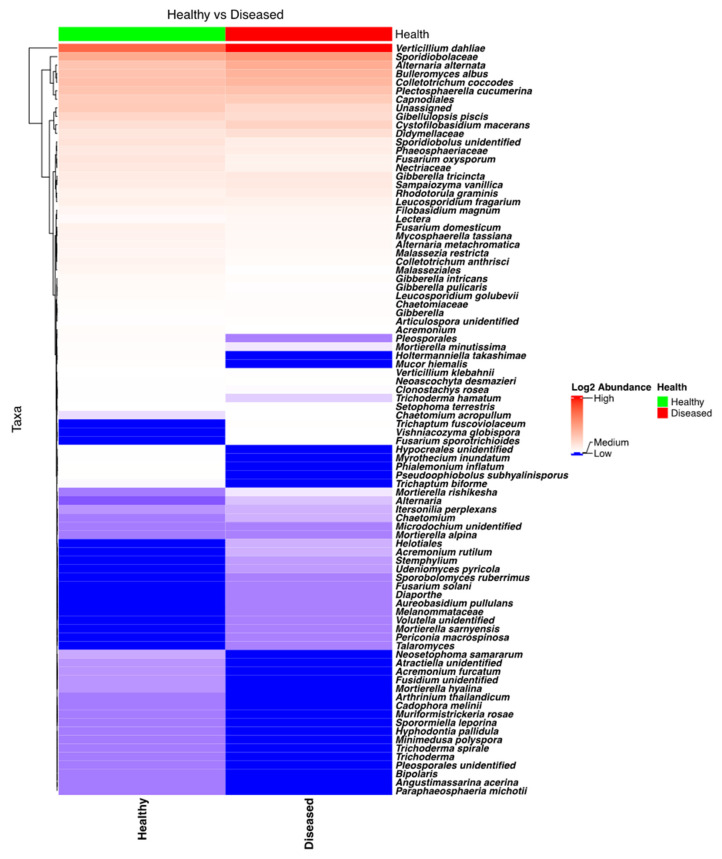
Heatmap of the fungal taxa found in the potato stems of diseased- and healthy-looking plants. NGSI data.

NGSI targeting ITS sequences identified a plethora of fungi, including several pathogenic to potatoes. Taxa from the genera *Verticillium*, *Plectosphaerella*, *Colletotrichum*, *Alternaria*, *Gibellulopsis*, and *Fusarium* were found to have high relative frequencies in the sequenced samples. Analysis of the heatmap (Figure 5) and the bar plot (Figure S7) revealed that *Verticillium* was extremely abundant in the stem samples coming from diseased plants, while its abundance was much lower in the stems of healthy plants. Specifically, *Verticillium*’s abundance in stems from healthy and diseased plants was found to be different, and this difference was highly significant (*p* < 0.0001). Among other potato pathogens that were found to be more abundant in symptomatic potato plants were *A. alternata* and *C. coccodes*. Detailed analysis revealed that the rotating crops (barley > oat) and location had strong influences on the abundance of *Verticillium* (*V. dahliae*) in the stems.

NGSI allowed the identification of a total of 88 ASVs. On average, 15.3 ± 3.8 ASVs were found in the stems of diseased-looking plants (range: 10–25 ASVs/stem) and 16.1 ± 5.0 in the stems of healthy-looking plants (range: 9–27 ASVs/stem).

**Figure 6:**
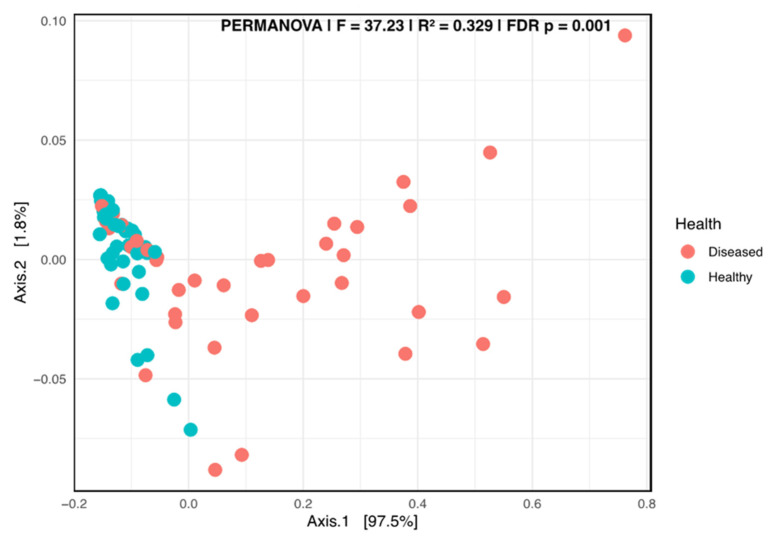
Principal coordinate analysis (PCoA) using the weighted Unifrac of the fungal taxa composition in the stems of diseased- and healthy-looking potato plants (taxonomic level 6). NGSI data.

A Kruskal–Wallis pairwise comparison test on the Shannon alpha diversity indicated that significant differences occurred between the healthy and diseased groups (*p* = 0.00452). The beta diversity plot, generated using the weighted UniFrac distance metric, revealed a clear separation of the two groups, suggesting a strong differentiation related to the health status of the plant (Figure 6).

**Figure 7:**
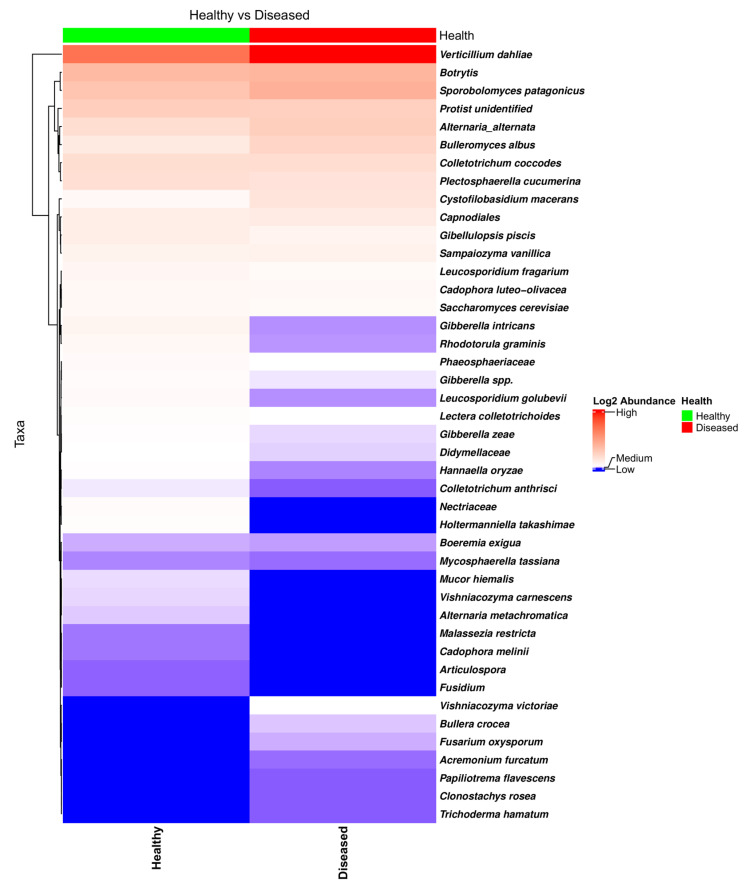
Heatmap of the fungal taxa found in the potato stems of diseased- and healthy-looking plants. NGSPB data.

NGSPB allowed the clear identification of the fungal components in the stem with a greater level of confidence, in many cases at the species level. This was one of the main goals in using the NGSPB technology. As shown in the heatmap (Figure 7) and bar plot (Figure S8), *V. dahliae* was clearly the most abundant pathogenic fungus, and its abundance was much higher in most diseased-looking plants. In addition to *V. dahliae*, some stems from diseased-looking plants had large amounts of *C. coccodes*, *Botrytis* sp., and *A. alternata*. *P. cucumerina* was found to be quite abundant in the stems of a few healthy-looking plants that contained much lower levels of *V. dahliae* (Figure 7). edgeR revealed a different abundance of *P. cucumerina* in fields in which the rotation crop was barley (FDR = 0.0007), being more abundant in diseased-looking plants. As mentioned in Section 3.2.1., edgeR indicated that, overall, *V. dahliae* was more abundant in the diseased-looking plants; however, this was true in the overall analyses and for the fields that were previously cultivated with oats (FDR = 0.0007) but not barley (FDR = 0.9347) fields, in which the abundance of *P. cucumerina* was found to be significantly different. The heatmap of the taxa identified using NGSPB (Figure 7) shows clearly that *V. dahliae* was the fungus with the highest incidence and relative abundance. *Botrytis* spp., *Sporobolomyces patagonicus*, *A. alternata*, *P. cucumerina*, *C. coccodes*, and an unidentified protist were found to have either a large incidence or high relative abundance in a number of samples.

NGSPB allowed the identification of 42 ASVs, less than half of the number identified by NGSI, a reflection of the depth of the sequencing. On average, 8.7 ± 3.2 ASVs were found in the stems of diseased-looking plants (range: 4–16 ASVs/stem) and 8.2 ± 6.1 ASVs in the stems of healthy-looking plants (range: 3–20 ASVs/stem). This result was similar to that obtained via NGSI; that is, there were no significant differences in the number of ASVs, and potentially different species, in the stems of diseased- and healthy-looking plants.

The Shannon alpha diversity was found to be significantly different when the healthy and diseased groups were compared (*p* = 0.03793). Similar to the NGSI data, the NGSPB dataset also revealed a distinct separation between the two groups in the beta diversity plot, indicating a strong differentiation associated with plant health status.

#### 3.2.4. Fungal Network Co-Occurrence and Differential Structure Related to Plant Health Status

**Figure 8:**
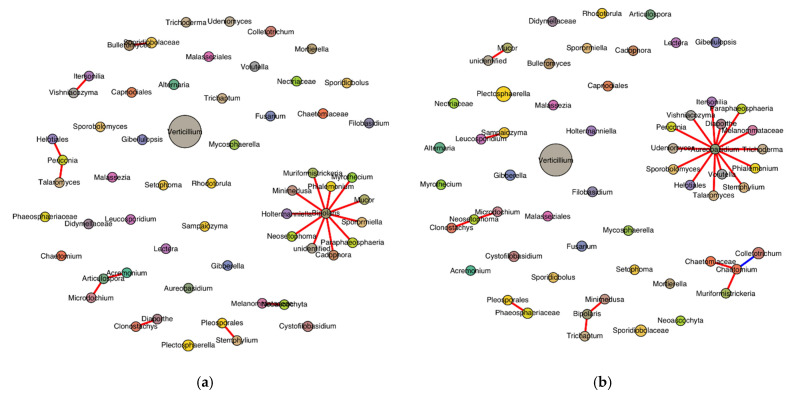
Genus-level SPIEC-EASI network in stems of healthy- (**a**) and diseased-looking plants (**b**). Node size reflects relative abundance, genus-specific colors differentiate nodes, and edge color represents the sign of the association (red for positive and blue for negative).

SPIEC-EASI successfully inferred the genus-level fungal interaction networks for both the healthy- and diseased-looking potato stem samples. Each network included 59 genera, indicating consistent taxonomic richness across conditions (Figure 8). The healthy network consisted of 24 edges, compared with 20 in the diseased network, reflecting a slightly higher density (0.014 vs. 0.012) and average node degree (0.81 vs. 0.68) in the healthy samples. Only the healthy network exhibited a negative edge (*n* = 1), while all edges in the diseased network were positive. The modularity score, used to evaluate community structure, was slightly higher in the diseased network (0.665) compared with the healthy network (0.625), suggesting more defined sub-community separation in diseased conditions. The Jaccard index for genus overlap was 1.0, confirming that all genera present in one network were also found in the other. Degree centrality analysis revealed distinct hub genera. In the healthy network, *Aureobasidium* had the highest connectivity (degree = 14), followed by *Chaetomium*, *Bipolaris*, *Neosetophoma*, and *Chaetomiaceae*. In contrast, the diseased network was dominated by *Bipolaris* (degree = 11), followed by *Articulospora*, *Periconia*, *Acremonium*, and *Bulleromyces*.

**Figure 9:**
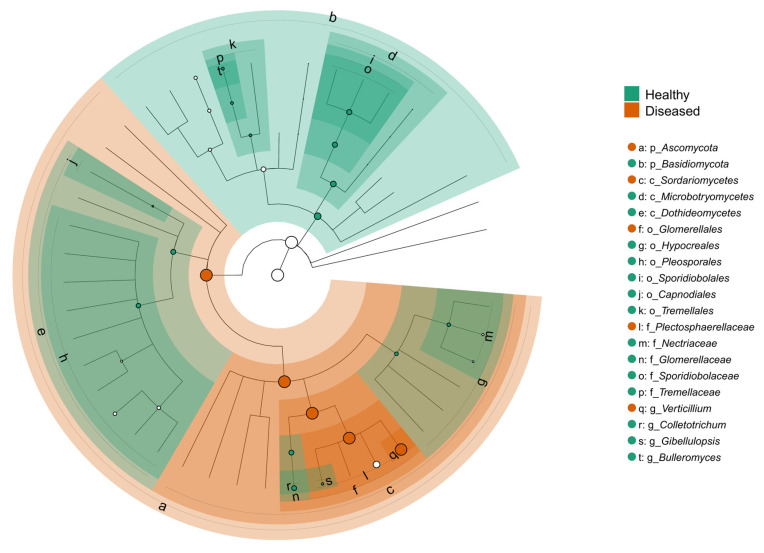
LEfSe cladogram comparing fungal taxa significantly enriched in the stems of healthy- and diseased-looking plants. The circular cladogram represents the taxonomic hierarchy from phylum (innermost ring) to genus and species (outermost tips). Green highlights indicate taxa significantly enriched in healthy stems, while orange represents taxa enriched in diseased stems (LDA score > 2.0, *p* < 0.05). Each lettered node corresponds to a taxonomic rank listed in the legend to the right.

The LEfSe cladogram revealed distinct taxonomic shifts in the fungal communities associated with the health status of potato plants (Figure 9). Taxa significantly enriched in the stems of healthy-looking plants were widely distributed across multiple taxonomic groups and included members of the orders *Pleosporales* and *Tremellales* and families such as *Tremellaceae* and *Glomerellaceae*. Genera like *Colletotrichum*, *Bulleromyces*, and *Sporidiobolus* were more abundant in healthy tissues. Conversely, the stems of diseased-looking plants showed enrichment in fungal taxa largely concentrated within the *Sordariomycetes* class. The most prominent genus associated with diseased treatments was *Verticillium*, and the species was *V. dahliae*. The order *Glomerellales* was also significantly enriched in the diseased-looking samples. The phylogenetic structure of the cladogram revealed that these health-associated taxa cluster within different fungal lineages, indicating that the disease state is accompanied not only by compositional shifts but also by changes in phylogenetic lineage. These results suggest that the fungal communities of potato stems respond to disease pressure in a structured and condition-specific manner, with potential implications for biological control or diagnostic biomarkers.

## 4. Discussion

*V. dahliae* was considered to be the main pathogen responsible for the occurrence of potato early dying disease in most regions in the USA, while *V. albo-atrum* was considered to be the dominant species in cooler production areas [2,32]. The same trend was found by studies carried out more than two decades ago in the Maritime region of Canada, in which two provinces, New Brunswick and Prince Edward Island, are major producers of potatoes; *V. albo-atrum* was reported to be the dominant species over *V. dahliae* [33,34,35,36]. The presence of *V. dahliae* in the region was reported for the first time in 1987 [33]. However, recent studies revealed that *V. dahliae* is the dominant species in the area [4,5,6]. These studies also highlighted the fact that in most fields used for potato production, *V. dahliae*‘s incidence and abundance are high. Moreover, root-lesion nematodes, such as *Pratylenchus crenatus* and *P. penetrans,* are also abundant in the production areas, with the former species having a much higher incidence [6]. However, little is known about the presence of other potato pathogens that have been suggested as contributing to the more severe symptoms of PED [1,2,37]. All recent studies were conducted using either classical methods such as pathogen counting from soil samples or via different PCR methods. Clearly, a different approach was needed to characterize the diversity of soil microbiota found in the proximity of potato plants and colonizing potato plants which can either enhance or suppress PED symptoms. By using NGSI and NGSPB, this study aimed to cover this gap in understanding the complexities of PED.

A comparison of the bacterial, fungal, and eukaryotic diversity and abundance in soil samples collected from the proximity of healthy and diseased plants revealed small differences; only the bacterial alpha diversity was found to be influenced by the health status of the plant. *V. dahliae* was found to be abundant in the soil samples collected from the proximity of healthy-looking plants as well as the proximity of diseased-looking plants, and no significant differences could be determined, suggesting that, overall, soil microbial communities had no significant suppressive effects on the pathogen. Analyses of the soil and plant samples confirmed what previous studies revealed; that is, *V. dahliae* is omnipresent in the area. Therefore, all fields analyzed could be considered conducive to *V. dahliae* and, likely, to PED. At the time of sampling, in early September, both the healthy- and diseased-looking potato plants were found to be infected by the pathogen. This finding was not entirely surprising because, in previous studies, many of the samples taken from healthy-looking plants have been found to be infected by *V. dahliae* [4,5]. *V. dahliae* from a natural inoculum was also found to successfully infect plants inoculated with *V. nonalfalfae* strain P1856, indicating that different *Verticillium* species are not mutually exclusive [19]. It is worth noting that qPCR and NGS analyses of *V. dahliae*‘s incidence and abundance in soil samples revealed no significant differences between those collected near healthy-looking and diseased-looking plants across all sampled fields. Additionally, no correlation could be established between the *V. dahliae* levels in the soil and stem samples. Therefore, relying solely on qPCR analysis of soil samples is unlikely to reliably predict the occurrence of PED.

This study found a large number of fungi present in the proximity of potatoes, including *Botrytis* spp., *A. alternata*, *C. coccodes*, *P. cucumerina*, and various species of *Fusarium* (e.g., *F. oxysporum*, *F. graminearum*, and *F. solani*), while common soil fungal saprotrophs, such as *Mortierella* and *Trichoderma*, have been found to be particularly abundant. Many of the species found in soil have also been identified in plants, including *Botrytis* spp., *A. alternata*, *P. cucumerina,* and *C. coccodes.* Interestingly, one taxon that is less known, *S. patagonicus*, was found to occur with a high incidence and be quite abundant in plants, though its presence in the soil samples was not noticeable. Though it was expected that we would find several fungal taxa in the potato stems, their diversity was quite surprising, with an average of 15.6 ASVs/stem identified in the NGSI data and 8.5 ASVs/stem identified in the NGSPB data. Though analyses of the fungal interaction networks failed to uncover specific interactions involving *V. dahliae*, differential abundance analysis using LEfSe revealed that the health status of the plants caused shifts in the fungal communities located in the stems.

Heatmap and edgeR analyses of the NGSI data revealed a large number of fungi that had a distribution or abundance that was significantly different in the healthy- and diseased-looking plants. Though not particularly abundant, it was surprising that *F. oxysporum*, which can cause fusarium wilt in a wide range of species, including in the Solanaceae family [32], was found to be statistically (FDR = 0.00085) more abundant in the healthy-looking plants. *G. piscis* was also found to be slightly more abundant in the healthy-looking plants, but this difference was statistically significant (FDR = 0.0127). *G. piscis* is pathogenic to various plant species [31], but attenuated strains were also tested for their potential antagonistic activity against *V. dahliae*, including in potatoes [38].

Species like *P. cucumerina*, *Bulleromyces albus*, and *Cystofilobasidium macerans* were found to be quite abundant in most stem samples, irrespective of health status. *P. cucumerina* is considered to pose a serious threat to plants from the Cucurbitaceae family [39,40,41] but can affect species from the Rubiaceae [42] and Ranunculaceae [41] families. On the other hand, the nematophagous capacity of this species was evaluated as a potential biological control against nematodes affecting potatoes [43]. Not much is known about the roles of *Bulleromyces albus* (teleomorph for *Bullera alba*), but there is some evidence that these taxa can secrete potent toxins with inhibitory effects for a range of ascomycetes and basidiomycetes [44]. *Cystofilobasidium macerans* was found to be an epiphyte on apples [45], and isolated strains have high cellulolytic activity [46]. Pathogenic species like *A. alternata* and *C. coccodes*, which cause potato brown spot disease and potato black dot disease, respectively, were found to be abundant in most stem samples, particularly in the diseased-looking plants (>3 times), but when using edgeR, this difference was not found to be statistically significant. The heatmap and edgeR indicated that two species, *Holtermanniella takashimae* and *Mucor hiemalis*, were quite specific to the diseased-looking plants, though these taxa were identified based on relatively large number of amplicons in a rather low number of samples (two and three, respectively). *Holtermanniella* species, including *H. takashimae*, are found in various substrates, including plants and soil [47]. *H. takashimae* was also found in the NGSPB data. *M. hiemalis* is not a particularly well-characterized taxon, but it was tested as a biocontrol agent due to its antagonistic abilities in soil and plant surfaces [48,49]. The presence of this taxon was not confirmed in the dataset generated by NGSPB.

NGSPB confirmed the presence of the most abundant taxa in the NGSI dataset. The only notable difference was the identification of *Botrytis* being abundant in most stem samples, without significant differences related to health status. Statistically, as mentioned in the Results section, only the abundance of *V. dahliae* was found to be different; *P. cucumerina*, *Gibberella intricans* (anamorph, *Fusarium equiseti*), and *Rhodotorula graminis* were found to be more abundant in the healthy-looking plants, but this difference was found to be borderline (FDR = 0.05). This difference was not observed in the case of *P. cucumerina* data generated by NGSI. The possible roles of *G. intricans* and *R. graminis* related to health status are not clear. *G intricans* causes wilt and root rot in a wide range of crops [50,51]. In contrast, a strain of *R. graminis*, isolated as an endophyte from poplar, has been found to improve plant vigor [52,53].

NGSI targeting 18S in soil samples revealed a wide range of eukaryotic communities that are relevant to better characterization of PED. This approach could be extremely useful for better understanding the nematode communities that are present in the fields used in potato production. Currently, nematode identification and quantitation involve significant work and can target a rather limited number of species [6]. In the current study, using NGSI, an extremely large number of diverse nematode genera were identified (*Pratylenchus*, *Ditylenchus*, *Filenchus*, *Helicotylenchus*, *Rhabditis*, *Cephalobus,* and *Distolabrellus*), many of which are important pathogens in potato production or for rotation crops.

While NGS data provided a clear picture of the fact that many species pathogenic to potatoes can synergistically trigger more severe symptoms, including PED, no specific correlations between or among the detected species could be revealed. Clearly, in the healthy-looking plants, *V. dahliae* was found to be less abundant. However, it cannot be determined if this was the result of suppressive interactions between species colonizing the xylem and surrounding tissues or a reflection of the timing of infection by *V. dahliae*. PED occurs or becomes most severe late in the growing season during the tuber-bulking period and early maturation [1,2]. Accordingly, *V. dahliae*‘s incidence and abundance are much lower in potato plants if qPCR assessments are performed in the Maritime region of Canada in early or mid-August (Borza, T., unpublished observations). To address these questions, more elaborate studies are needed, including a few time points before the occurrence of PED.

## 5. Conclusions

The current view is that *V. dahliae*, *V*. *nonalfalfae*, and *V*. *albo-atrum* can cause *Verticillium* wilt, which generally causes less severe symptoms than PED. To explain the difference in disease severity between *Verticillium* wilt and PED, it has been proposed that PED occurs as a result of the cumulative effects of several other pathogens, including soft-rot bacteria, fungi (such as *Colletotrichum* and *Fusarium*), and root-lesion nematodes (species from the genus *Pratylenchus*) [1,2,32,37]. The current study provides little support for this differentiation, as all healthy- and diseased-looking plants were found to be colonized by *V. dahliae*, and all displayed highly diverse microbiota, with complex consortia of pathogenic species, potentially antagonistic species, and taxa with roles that are not well understood. Though *V. dahliae* was found to be significantly more abundant in potato plants displaying severe symptoms, the results of this study indicate that several other bacteria and fungi, not only *Verticillium* species, colonize the plants in natural habitats, affecting their health status. It remains to be determined how plant colonization occurs, in what succession, and how the establishment of one species inhibits or facilitates the abundance of others. In the current study, NGSI showed that several bacteria and, on average, 15 different fungi can colonize the lower part of the stem. Additional studies targeting multiple parts of the plant and including several time points can further contribute to elucidating the complexities of PED.

## Figures and Tables

**Table 1:** *V. dahliae* (Vd) abundance and incidence in soil samples collected from the proximity of healthy- and diseased-looking potato plants, estimated using qPCR.

Field #	Rotation Crop in the Previous Year	Sample Type	Average Number of Vd Cells/g Soil	SE	Incidence
1	Oat	healthy	1104.5	370.1	70
		diseased	743.9	212.1	70
2	Oat	healthy	2511.3	1203.6	100
		diseased	2450.5	1288.8	90
3	Barley	healthy	1266.5	495.2	100
		diseased	1602.9	388.5	100
4	Barley	healthy	152.9	36.7	60
		diseased	1030.8	489.0	90

**Table 2:** *V. dahliae* (Vd) abundance and incidence in potato stem samples collected from healthy and diseased plants estimated using qPCR. * Significant difference between the means (*p* < 0.05; two-sample *t*-test).

Field #	Rotation Crop in the Previous Year	Sample Type	Average Number of Vd Cells x 10^3^/g Fresh Tissue	SE	Incidence
1	oat	healthy	23.8	7.3	100
		diseased	35.1	13.7	80
2	oat	healthy	51.7	14.4	100
		diseased	986.9	531.9	100
3	barley	healthy	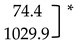	26.8	100
		diseased		430.5	100
4	barley	healthy	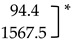	32.0	100
		diseased		364.7	100

## Data Availability

The original contributions presented in this study are included in the article/Supplementary Material. Further inquiries can be directed to the corresponding authors. Sequencing data have been deposited at the Sequence Read Archive (SRA), URL https://www.ncbi.nlm.nih.gov/sra/ (accessed on 18 June 2025); project ID PRJNA1263883, SRA study ID SRP585969.

## Supplementary Materials

The following supporting information can be downloaded at https://www.mdpi.com/article/10.3390/microorganisms13071482/s1: Figure S1. Healthy- and diseased-looking plants in the field. a. Image of the field showing a mix of healthy- and diseased-looking plants. b. A close up of a plant that was considered as showing clear symptoms of potato early dying (PED). Figure S2. Barplot of the bacterial taxa composition in soil samples collected from the proximity of diseased- and healthy-looking potato plants (taxonomic level 6). NGSI data. Figure S3. Principal coordinate analysis (PCoA) using weighted Unifrac of the bacterial taxa composition in soil samples collected from the proximity of diseased- and healthy-looking potato plants (taxonomic level 6). NGSI data. Figure S4. Barplot of the fungal taxa composition in soil samples collected from the proximity of diseased- and healthy-looking potato plants (taxonomic level 6). NGSI data. Figure S5. Principal coordinate analysis (PCoA) using weighted Unifrac of the fungal taxa composition in soil samples collected from the proximity of diseased- and healthy-looking potato plants (taxonomic level 6). NGSI data. Figure S6. Barplot of the eukaryotic taxa composition in soil sample collected from the proximity of diseased- and healthy-looking potato plants (taxonomic level 6). NGSI data. Figure S7. Barplot of the fungal taxa composition in soil and in the stems of diseased- and healthy-looking plants (taxonomic level 7). NGSI data. Figure S8. Barplot of the fungal taxa composition in soil and in the stems of diseased- and healthy-looking plants (taxonomic level 7). NGSPB data. Table S1. Primers used in Illumina MiSeq sequencing targeting short variable regions (https://imr.bio/protocols.html). Table S2. Primers used in PacBio Vega sequencing targeting entire regions (https://imr.bio/protocols.html). References [54,55,56,57] are cited in the supplementary materials.
