# Supplementary material for: Microbial Community Composition Associated with Potato Plants Displaying Early Dying Syndrome
Source: Microorganisms. 2025 Jun 26;13(7):1482. doi: 10.3390/microorganisms13071482 (PMC12300998; doi:10.3390/microorganisms13071482)
Supplement: Supplementary file 1 [file microorganisms-13-01482-s001.zip › Supplemental material PED_Figures_r1.pdf]

Supplementary Figures S1 – S8.

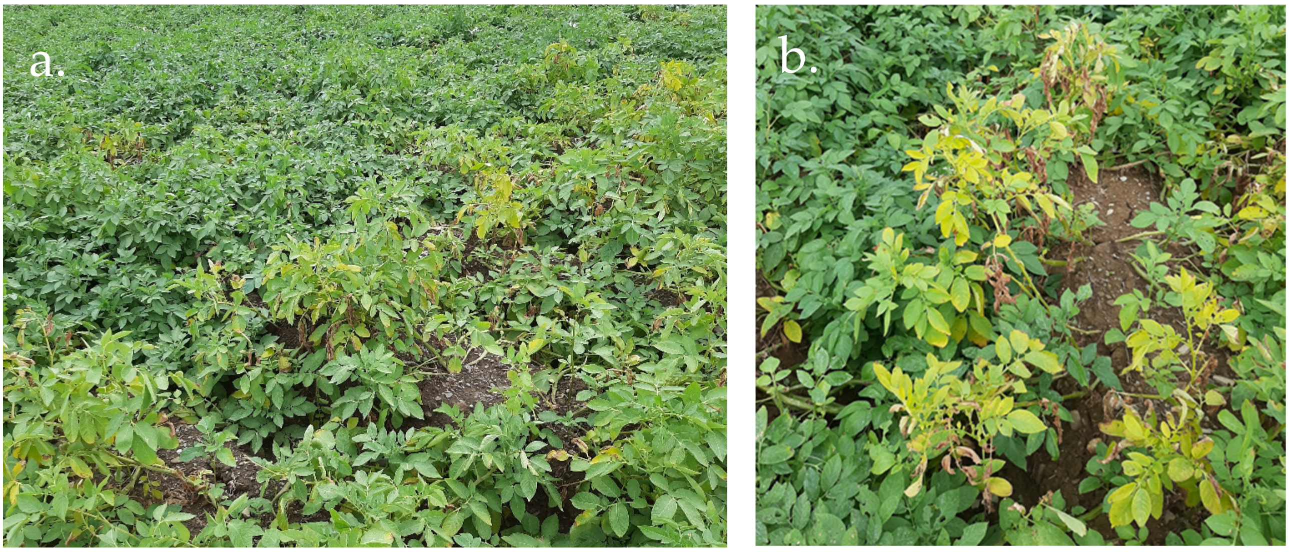

Figure S1. Healthy- and diseased-looking plants in the field. a. Image of the field showing a mix of healthy- and diseased-looking plants. b. A close up of a plant that was considered as showing clear symptoms of potato early dying (PED).

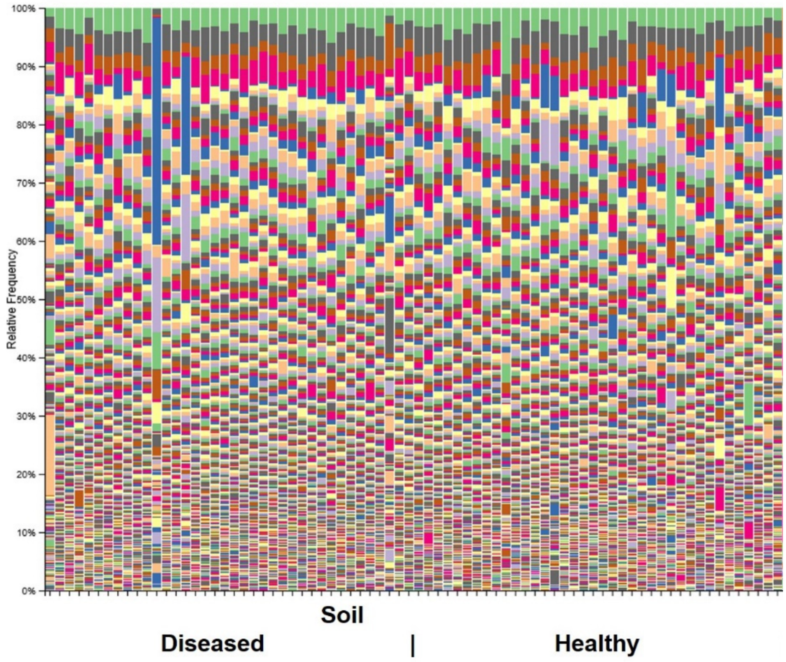

Figure S2. Barplot of the bacterial taxa composition in soil samples collected from the proximity of diseased- and healthy-looking potato plants (taxonomic level 6). NGSI data.

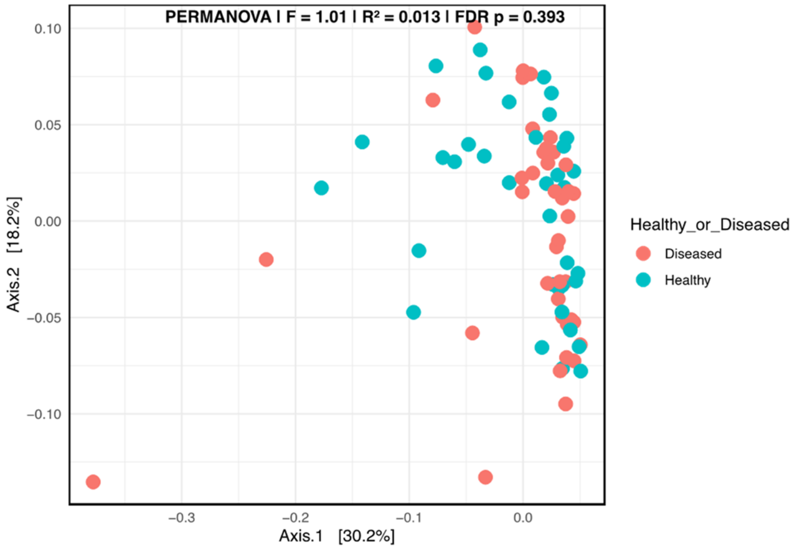

Figure S3. Principal coordinate analysis (PCoA) using weighted Unifrac of the bacterial taxa composition in soil samples collected from the proximity of diseased- and healthy-looking potato plants (taxonomic level 6). NGSI data.

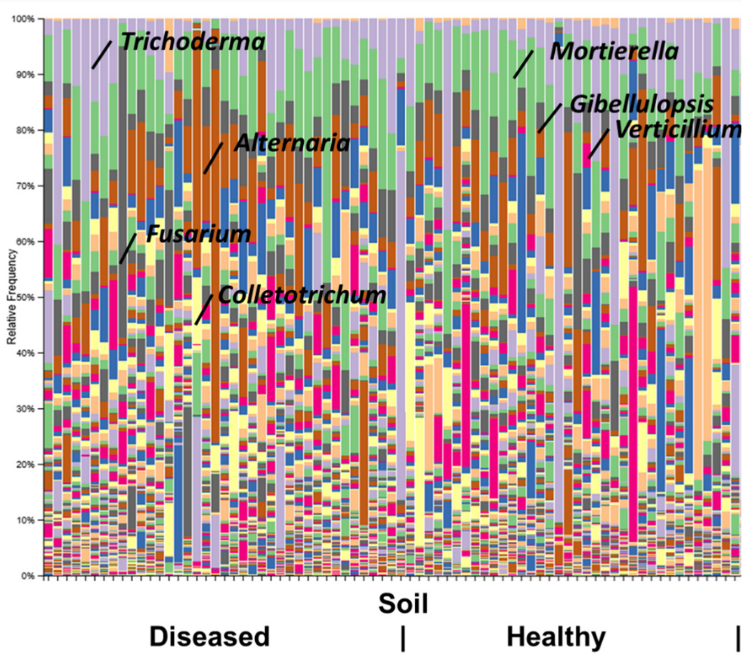

Figure S4. Barplot of the fungal taxa composition in soil samples collected from the proximity of diseased- and healthy-looking potato plants (taxonomic level 6). NGSI data.

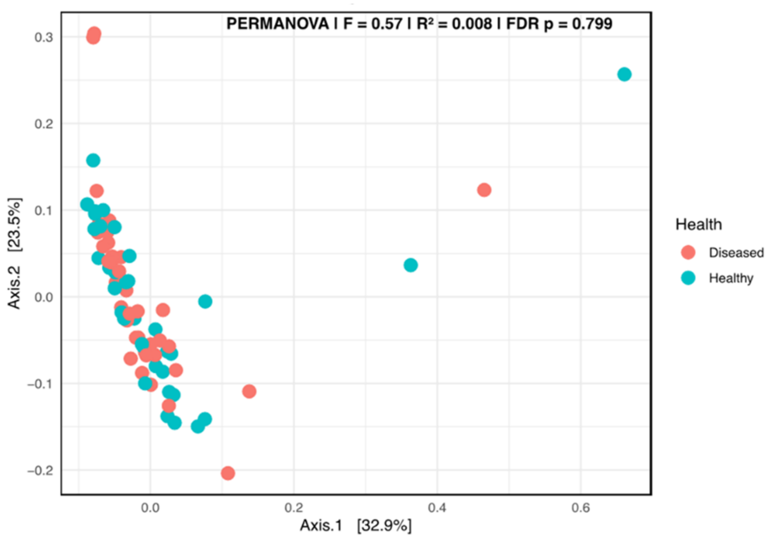

Figure S5. Principal coordinate analysis (PCoA) using weighted Unifrac of the fungal taxa composition in soil samples collected from the proximity of diseased- and healthy-looking potato plants (taxonomic level 6). NGSI data.

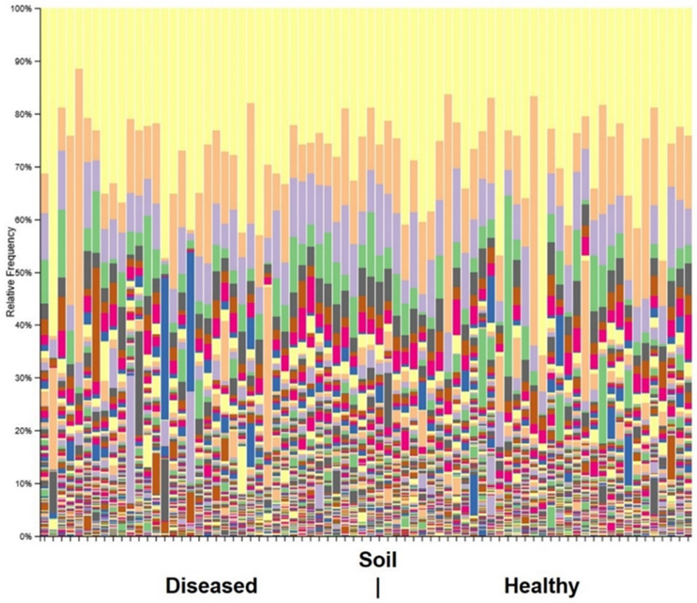

Figure S6. Barplot of the eukaryotic taxa composition in soil sample collected from the proximity of diseased- and healthy-looking potato plants (taxonomic level 6). NGSI data.

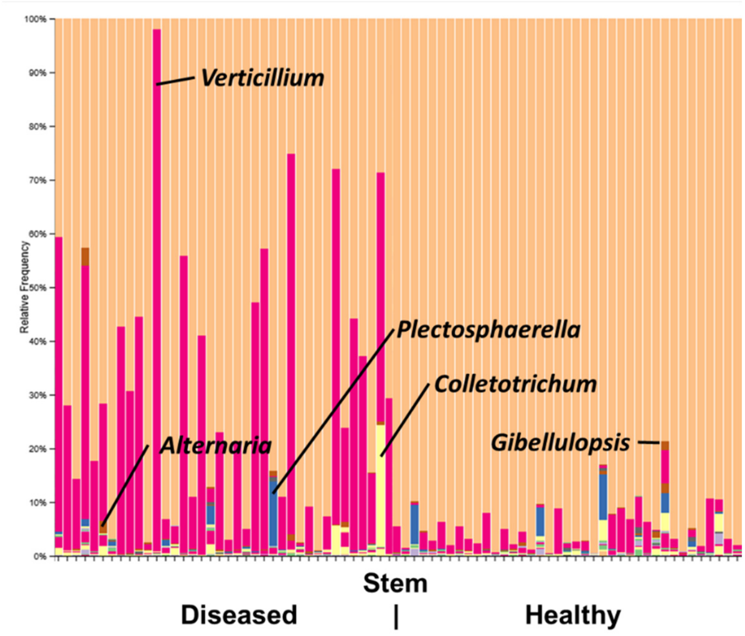

Figure S7. Barplot of the fungal taxa composition in soil and in the stems of diseased- and healthy-looking plants (taxonomic level 7). NGSI data.

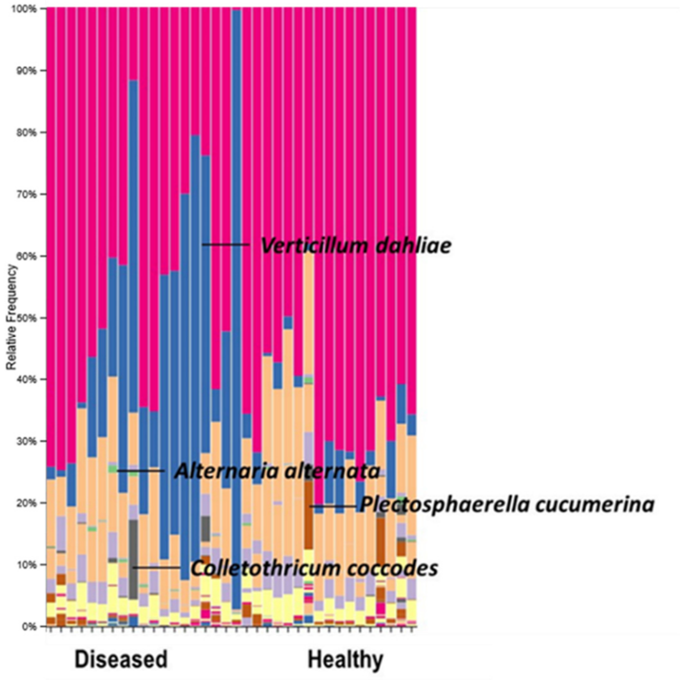

Figure S8. Barplot of the fungal taxa composition in soil and in the stems of diseased- and healthy-looking plants (taxonomic level 7). NGSPB data.
