# Supplementary material for: Microbial Community Composition Associated with Potato Plants Displaying Early Dying Syndrome
Source: Microorganisms. 2025 Jun 26;13(7):1482. doi: 10.3390/microorganisms13071482 (PMC12300998; doi:10.3390/microorganisms13071482)
Supplement: Supplementary file 1 [file microorganisms-13-01482-s001.zip › Supplemental material PED_Tables_r1.pdf]

Table S1. Primers used in Illumina MiSeq sequencing targeting short variable regions (https://imr.bio/protocols.html)

Taxonomic group/DNA targeted area	Primer sequence	References
Bacteria-specific (rRNA V6-V8)	B969F = ACGCGHNRAACCTTACC	[1]
	BA1406R = ACGGGCRGTGWGTRCAA	
Eukaryote-specific (rRNA V4)	E572F = CYGCGGTAATTCCAGCTC	[1]
	E1009R = AYGGTATCTRATCRTCTTYG	
Fungi-specific (ITS2)	ITS86(F) = GTGAATCATCGAATCTTTGAA	[2]
	ITS4(R) = TCCTCCGCTTATTGATATGC	

Table S2. Primers used in PacBio Vega sequencing targeting entire regions (https://imr.bio/protocols.html)

Taxonomic group/DNA targeted area	Primer sequence	References
Bacteria-specific (Full 16S)	27F(Paliy) = AGRGTTYGATYMTGGCTCAG	[3]
	1492R = RGYTACCTTGTTACGACTT	
Fungi-specific (Full ITS)	ITS1FKYO2 = TAGAGGAAGTAAAAGTCGTAA	[4]
	ITS4KYO1 = TCCTCCGCTTWTTGWTWTGC	

1. Comeau, A.M.; Li, W.K.W.; Tremblay, J.-É.; Carmack, E.C.; Lovejoy, C. Arctic ocean microbial community structure before and after the 2007 record sea ice minimum. *PLOS ONE* **2011**, *6*, e27492, doi:10.1371/journal.pone.0027492.
2. Op De Beeck, M.; Lievens, B.; Busschaert, P.; Declerck, S.; Vangronsveld, J.; Colpaert, J.V. Comparison and validation of some ITS primer pairs useful for fungal metabarcoding studies. *PLOS ONE* **2014**, *9*, e97629, doi:10.1371/journal.pone.0097629.
3. Paliy, O.; Kenche, H.; Abernathy, F.; Michail, S. High-throughput quantitative analysis of the human intestinal microbiota with a phylogenetic microarray. *Applied and Environmental Microbiology* **2009**, *75*, 3572-3579, doi:doi:10.1128/AEM.02764-08.
4. Toju, H.; Tanabe, A.S.; Yamamoto, S.; Sato, H. High-coverage ITS primers for the DNA-based identification of ascomycetes and basidiomycetes in environmental samples. *PLOS ONE* **2012**, *7*, e40863, doi:10.1371/journal.pone.0040863.
